# Supplementary material for: Crash severity analysis of vulnerable road users using machine learning
Source: PLoS One. 2021 Aug 5;16(8):e0255828. doi: 10.1371/journal.pone.0255828 (PMC8341492; doi:10.1371/journal.pone.0255828)
Supplement: Attachment — Submitted filename: Response to Reviewer.docx [file pone.0255828.s001.docx]

**Paper Title:** Crash Severity Analysis of Vulnerable Road Users using Machine Learning

**Response to the Reviewers’ Comments**

**Manuscript ID:** PONE-D-21-14520

We would like to thank the reviewers for their valuable comments. We believe that the comments have identified important areas that required improvement. We have addressed the comments point by point and revised the paper accordingly. Please note that our responses to the comments are highlighted in the context of the paper in a different colour or track changes.

**Reviewers’ comments:**

**#1 Submitted by: Reviewer 1**
**Comments:**

1. Among the various machine learning methods, why were the three algorithms, namely KNN, SVM, and RF, used for analysis? More justifications on the advancement of these three methods are required in the Introduction and Literature Review sections.

**Response:**

**The justification for using these models is added in the introduction section of the revised paper.**

So far, there is no specified rule to apply a particular machine learning model for a specific application [9]. Therefore, most of the research uses multiple machine learning models to the same dataset and use comparative analysis. For example, the study performed by Thanh Noi and Kappas [10] compared the performance of three supervised machine learning models, i.e., KNN, RFC and SVM, in the classification of remote sensing images. The authors found the SVM classifier to be the best performing model in this application, while considering accuracy as performance measure. This indicate that different machine learning models show promises in different applications; however, which classifier is best for a specific application is still not clear.

Specifically, in the existing road safety and crash severity analysis research, several studies have compared the performance between machine learning models where KNN, SVM, and RF models show their performance advantages at different domains [11-13]. Therefore, all of these models bear the significance to be used in machine learning-based analysis for road crash severity. These models perform better in different applications, and it is not feasible to conclude superiority of one without comparing their performance for the specific application.

Reference:

9.	Chen J, Wang H, Hua C. Assessment of driver drowsiness using electroencephalogram signals based on multiple functional brain networks. International Journal of Psychophysiology. 2018;133:120-30.

10.	Thanh Noi P, Kappas M. Comparison of random forest, k-nearest neighbor, and support vector machine classifiers for land cover classification using Sentinel-2 imagery. Sensors. 2018;18(1):18.

11.	Elamrani Abou Elassad Z, Mousannif H, Al Moatassime H. A real-time crash prediction fusion framework: An imbalance-aware strategy for collision avoidance systems. Transportation Research Part C: Emerging Technologies. 2020;118:102708.

12.	Zhang J, Li Z, Pu Z, Xu C. Comparing Prediction Performance for Crash Injury Severity Among Various Machine Learning and Statistical Methods. IEEE Access. 2018;6:60079-87.

13.	Fiorentini N, Losa M. Handling Imbalanced Data in Road Crash Severity Prediction by Machine Learning Algorithms. Infrastructures. 2020;5(7).

2. The discrete choice models have long been used for crash severity analysis (Mannering et al., 2016). More importantly, the random-parameter models have been demonstrated to be superior in accounting for the unobserved heterogeneity, with a substantial improvement in goodness-of-fit and interpretability (Chang et al., 2019; Chen et al., 2019; Waseem et al., 2019; Alogaili and Mannering, 2020; Wang et al., 2020; Zhou et al., 2020; Zhou et al., 2021). These methodological alternatives should not be ignored, particularly in the Literature Review section. In addition, since the random-parameter logit model has become the benchmark for crash data analysis (Mannering and Bhat, 2014), the authors are highly suggested to include this method for comparison, which may help to further highlight the advantages of machine learning methods.

**Response: We have added the following context and references in the literature review section to address the reviewer’s concern.**

**The suggested comparison between the discrete choice model and machine learning is beyond the scope of this study since our main focus was on comparing and contrasting effects of factors on different vulnerable road user groups safety by employing a machine learning approach. However, we appreciate the kind suggestions, and we have added the suggested comparison as a future research avenue in the conclusion section of the revised manuscript.**

Discrete choice model, such as random parameter model, random parameter ordered probit analysis, random parameter logit model, mixed logic model are most common statistical approaches used in different road crash data analysis [23-33].

References:

23.	Dong B, Ma X, Chen F. Analyzing the Injury Severity Sustained by Non-Motorists at Mid-Blocks considering Non-Motorists’ Pre-Crash Behavior. Transportation Research Record. 2018;2672(38):138-48.

24.	Shao X, Ma X, Chen F, Song M, Pan X, You K. A Random Parameters Ordered Probit Analysis of Injury Severity in Truck Involved Rear-End Collisions. International Journal of Environmental Research and Public Health. 2020;17(2).

25.	Alogaili A, Mannering F. Unobserved heterogeneity and the effects of driver nationality on crash injury severities in Saudi Arabia. Accident Analysis & Prevention. 2020;144:105618.

26.	Chang F, Xu P, Zhou H, Chan AHS, Huang H. Investigating injury severities of motorcycle riders: A two-step method integrating latent class cluster analysis and random parameters logit model. Accident Analysis & Prevention. 2019;131:316-26.

27.	Chen F, Song M, Ma X. Investigation on the Injury Severity of Drivers in Rear-End Collisions Between Cars Using a Random Parameters Bivariate Ordered Probit Model. International Journal of Environmental Research and Public Health. 2019;16(14).

28.	Mannering FL, Bhat CR. Analytic methods in accident research: Methodological frontier and future directions. Analytic Methods in Accident Research. 2014;1:1-22.

29.	Mannering FL, Shankar V, Bhat CR. Unobserved heterogeneity and the statistical analysis of highway accident data. Analytic Methods in Accident Research. 2016;11:1-16.

30.	Wang J, Huang H, Xu P, Xie S, Wong SC. Random parameter probit models to analyze pedestrian red-light violations and injury severity in pedestrian–motor vehicle crashes at signalized crossings. Journal of Transportation Safety & Security. 2020;12(6):818-37.

31.	Waseem M, Ahmed A, Saeed TU. Factors affecting motorcyclists’ injury severities: An empirical assessment using random parameters logit model with heterogeneity in means and variances. Accident Analysis & Prevention. 2019;123:12-9.

32.	Zhou H, Yuan C, Dong N, Wong SC, Xu P. Severity of passenger injuries on public buses: A comparative analysis of collision injuries and non-collision injuries. Journal of Safety Research. 2020;74:55-69.

33.	Zichu Z, Fanyu M, Cancan S, Richard T, Zhongyin G, Lili Y, et al. Factors associated with consecutive and non-consecutive crashes on freeways: A two-level logistic modeling approach. Accident Analysis & Prevention. 2021;154:106054.

**In the Discussion and Limitation section-**

“It might be beneficial to compare the performance of discrete choice models with the selected machine learning approaches of our study as a future research avenue.”

3. When investigating the safety of vulnerable road users, the type of collided vehicles (e.g., private cars, vans, trucks or buses) is expected to have a predominant effect on crash severities. Such information is readily available in the police records and should thus be included for model specification.

**Response: For our study, we focused only on Queensland road crash data provided by the Department of Transport and Main Road (Queensland). We have added your suggested idea in the Discussion and Limitation Section of the updated manuscript.**

“For our analysis, we only consider the dataset of road crash data provided by Department of Transport and Main Road (Queensland). However, the type of collided vehicles (e.g., private cars, vans, trucks or buses) may have a predominant effect on crash severities of vulnerable road users. Such information can be available in the Queensland police records and can be included for the further improvement of crash severity model specification if available.”

4. There are typos throughout the manuscript. For example, on page 9 line 194, “this help understanding…” should be “this helps in understanding…”; on page 10 line 221, “bicyclist” and “pedestrian” should be “bicyclists” and “pedestrians”; on page 24 line 286, “four different classifiers” should be “three different classifiers”.

**Response: Thanks a lot for indicating the corrections. We proofread the whole document and revised the manuscript carefully in addressing the concerns. Also, we have corrected Figure 2, which had some typos before.**

**#2 Submitted by: Reviewer 2**

**Comments:**

1. for injury severity studies, the choice between statistical models and machine learning ones is often a tradeoff between model interpretation and predictive performance. The authors should add discussion about the interpretability of the chosen machine learning models.

**Response: We appreciate your kind comment. We have added the following context to discuss model architecture in section Classification Approaches.**

Most of the statistical models work to infer the relationship between or among two or more variables, whereas the machine learning models deal with making the best accurate predictions [50]. The predictor outcomes are used to identify the class of the target variables. On the one hand, the statistical models can handle very small datasets, whereas the machine learning models are designed to deal with big data [51]. The dataset contains 21158 data points, having 17 features. Using statistical methods, such as ANOVA F Test or Correlation-based methods, could make the interpretation complex; thus, we have used machine learning models in this study. Given the different machine learning-based predictive models works on their specific principles, it is worth comparing their performances on the same dataset. The three selected models are supervised learning algorithms. SVM is a linear model that identifies the best classification hyperplane to separate data into desired classes [52]. KNN algorithm analyses the similarity of the data to classify different classes [53]. It considers a K value at the nearest neighbour to the data point for classification. The random forest acts as an ensemble method bagging multiple decision trees for classification [54]. The decision tree is an algorithm containing series of trees with the binary decision about the class. As such, random forest outperforms decision tree as an ensemble technique, decision tree model is not separately used for our crash severity analysis.

References:

50.	Tang J, Zheng L, Han C, Yin W, Zhang Y, Zou Y, et al. Statistical and machine-learning methods for clearance time prediction of road incidents: A methodology review. Analytic Methods in Accident Research. 2020;27:100123.

51.	Makridakis S, Spiliotis E, Assimakopoulos V. Statistical and Machine Learning forecasting methods: Concerns and ways forward. PLOS ONE. 2018;13(3):e0194889.

52.	Cristianini N, Ricci E. Support Vector Machines. In: Kao M-Y, editor. Encyclopedia of Algorithms. New York, NY: Springer New York; 2016. p. 2170-4.

53.	Peterson LE. K-Nearest Neighbour. Scholarpedia. 2009;4(2).

54.	Pal M. Random forest classifier for remote sensing classification. International Journal of Remote Sensing. 2005;26(1):217-22.

2. The literature review should be strengthened, the following relevant severity studies should be acknowledged and discussed in the paper:

[1] Shao Xiaojun, Ma Xiaoxiang, Chen Feng, Song Mingtao, Pan Xiaodong, You Kesi, 2020. A random parameters ordered probit analysis of injury severity in truck involved rear-end collisions. International journal of environmental research and public health. doi:10.3390/ijerph17020395

[2] Dong Bowen, Ma Xiaoxiang, Chen Feng, 2018. Analyzing the Injury Severity Sustained by Non-motorists at Mid-block Considering Non-motorists’ Pre-crash Behavior, Transportation Research Record.

**Response: Thanks for the comment. We have added these references in literature review section and the following context was added.**

Discrete choice model, such as random parameter model, random parameter ordered probit analysis, random parameter logit model, mixed logic model are most common statistical approaches used in different road crash data analysis [23-33].

References:

23.	Dong B, Ma X, Chen F. Analyzing the Injury Severity Sustained by Non-Motorists at Mid-Blocks considering Non-Motorists’ Pre-Crash Behavior. Transportation Research Record. 2018;2672(38):138-48.

24.	Shao X, Ma X, Chen F, Song M, Pan X, You K. A Random Parameters Ordered Probit Analysis of Injury Severity in Truck Involved Rear-End Collisions. International Journal of Environmental Research and Public Health. 2020;17(2).
